# Global high-resolution ultrafine particle number concentrations through data fusion with machine learning

**DOI:** 10.1038/s41597-025-06055-9

**Published:** 2025-11-14

**Authors:** Pantelis Georgiades, Matthias Kohl, Mihalis A. Nicolaou, Theodoros Christoudias, Andrea Pozzer, Constantine Dovrolis, Jos Lelieveld

**Affiliations:** 1 Computation-based Science and Technology Research Centre (CaSToRC), The Cyprus Institute, Nicosia, Cyprus; 2 Climate and Atmosphere Research Center (CARE-C), The Cyprus Institute, Nicosia, Cyprus; 3 Department of Atmospheric Chemistry, Max Planck Institute for Chemistry, Mainz, Germany

**Keywords:** Climate sciences, Atmospheric science

## Abstract

Atmospheric pollution causes millions of excess deaths annually, with particulate matter (PM) being a major concern. While research has traditionally focused on PM_10_ and PM_2.5_, ultrafine particles (UFPs, diameter < 100 nm) have emerged as a critical human health risk due to their ability to penetrate deeply into the respiratory system, transmigrate into the bloodstream and induce systemic health impacts. The total particle number concentration (PNC) serves as a proxy measure for UFP prevalence, as UFPs dominate particle number counts despite contributing minimally to total particle mass. This study presents the first global datasets of PNCs and UFPs at 1 km resolution over land by combining ground station measurements with machine learning. We developed an XGBoost model to predict annual PNC levels from 2010–2019, integrating diverse environmental and anthropogenic variables available at the global scale. Our model achieves an R^2^ of ≥0.9 and a mean relative error of about 30% for polluted urban areas, based on comparison with test datasets, and its performance was evaluated by including spatial and temporal cross-validation schemes. We find that global annual mean PNCs near the Earth’s surface vary between a few thousand per cm^3^ in pristine environments up to more than 40,000 per cm^3^ in some urban centres and that UFPs contribute about 91% to PNCs. The model incorporates a conformal prediction framework to provide reliable coverage intervals, making local-to-global PNC and UFP data available and supporting exposure assessments and health impact studies.

## Background & Summary

The growing concern surrounding atmospheric pollution stems from its well- established, detrimental impacts on human health^1^. Recent estimates suggest that air pollution is responsible for many millions of excess deaths annually and a leading contributor to the loss of healthy years of life^2,3^. Particulate matter (PM), a diverse category of airborne pollutants, consists of minute particles of solids and liquids suspended in the air, classified based on their aerodynamic diameter. Although historical evidence has long underscored the risks associated with PM exposure, recent global trends have amplified these concerns^4,5^. The growing population with intensifying industrialization, urbanization, as well as agricultural emissions, have collectively led to a substantial increase in atmospheric PM levels^6^.

Until recently, the emphasis was predominantly on particulate matter (PM) with diameters less than 10 *μ*m (PM_10_) and 2.5 *μ*m (PM_2.5_), often referred to as *coarse* and *fine* particulate matter, respectively^7,8^. Prolonged exposure to enhanced concentrations of these particles has been demonstrated to exert adverse effects on the respiratory and cardiovascular systems. Both PM_10_ and PM_2.5_ affect the respiratory tract, with the smaller particles generally penetrating more deeply into the lungs, and long-term exposure causes inflammation and oxidative stress, associated with enhanced disease risk, leading to chronic obstructive pulmonary disease (COPD), asthma, lung cancer, strokes, and heart attacks^9,10^.

There is growing concern about the health implications of PM smaller than PM_2.5_. At the lower end of the size distribution, ultrafine particles (UFPs) are those with an aerodynamic diameter less than 0.1 *μ*m or 100 nm (PM_0.1_), a subset of PM_2.5_^11^. Despite constituting a minor proportion of PM_2.5_ by mass, UFPs dominate in terms of number concentrations. In fact, the total particle number concentration (PNC) is often employed as a proxy measure for the UFP prevalence^12^. Natural sources of UFPs include new particle formation from inorganic and organic gases emitted by marine and forest ecosystems. The main sources of UFPs relevant to health, though, are anthropogenic and related to the use of fossil and biofuels, such as oil and coal combustion, notably from vehicular, marine and air traffic, energy generation, and various industrial sources^13^.

The small size of UFPs facilitates deep infiltration into the respiratory system, allowing them to reach the alveoli, transmigrate into the bloodstream and thereby cause adverse health effects in the vasculature and distant organs^14^. The large number combined with the and large surface-to-mass ratio of UFPs may promote interactions with biological tissue, potentially instigating inflammatory responses and oxidative stress. These molecular interactions have been implicated in several health conditions, including respiratory and cardiovascular diseases, as well as carcinogenesis^15^. Furthermore, recent epidemiological studies in New York and major cities in Canada have identified links between long-term exposure to UFPs and increases in non-accidental mortality in adults and children^16,17^.

Fine-grained maps of UFP concentrations are necessary for epidemiological assessments aiming at unravelling relationships between air pollution and public health outcomes^18^. High-resolution mapping enables researchers to conduct detailed spatial analyses, identify vulnerable populations, and understand the complex interplay between environmental factors and health. Such maps are fundamental for policymakers to formulate targeted interventions and regulatory policies to reduce UFP exposure and mitigate associated health risks effectively^19^.

The investigation of UFPs and their impact on human health is hindered by the scarcity of measurements, especially at the global scale. Existing monitoring systems lack the spatial coverage necessary for a comprehensive understanding of UFP distributions and determining long-term exposure. Furthermore, the intricate nature of UFPs, characterized by their small size and dynamic behaviour, poses challenges for traditional measurement techniques^20^. The recent literature on estimating the long-term mean, spatially distributed UFP concentrations largely depends on two main methodologies: land use regression models and chemical transport models. Each of these approaches, however, comes with limitations that impact their effectiveness in various contexts.

Land use regression models are known for their ability to provide high spatial resolution, making them particularly useful for detailed local analyses. However, their utility is confined to specific geographic regions with good coverage of UFP measurements. The reliance on local data and the necessity for model training procedures to be tailored to the particularities of each area was highlighted in studies by Saha (2021) and Jones (2020)^21,22^. Such dependence on localized data sources and custom training means that extending these models beyond their original scope can be challenging. Moreover, LUR model accuracy for UFPs is typically moderate, with explained variance (*R*^2^) ranging from 0.38 to 0.66 across different study areas, and cross-validation performance often 8–11% lower than model *R*^2^ values^22,23^. External validation studies demonstrate *R*^2^ values of approximately 0.50–0.53 when applied to independent datasets, with root mean square errors ranging from 2,800 to 3,500 particles/*cm*^−3^^23^. The transferability of LUR models to new geographic regions remains limited, with substantial reductions in explained variance when models are applied beyond their training domains^24^.

Chemical transport models extend an option to extend the geographical coverage, as they are designed to achieve broader spatial extent up to global applicability. However, this extensive coverage comes at the cost of spatial resolution due to computational constraints. Typically, these models operate at coarse resolution, in the range of 10 to 100 kilometres^25^. While recent advances have enabled some regional CTMs to reach spatial resolutions as fine as 3-5 km, or even sub-kilometer scales in limited applications^26,27^, substantial uncertainties persist in UFP prediction. CTMs are inherently limited by uncertainties in emission inventories, nucleation and coagulation parameterizations, meteorological inputs, and chemical mechanism representations^26^. These challenges often result in moderate model performance, with correlation coefficients typically ranging from 0.40 to 0.82 when validated against observational data, and systematic biases that vary by season and location^27^. The models frequently struggle to resolve steep spatial gradients in UFP concentrations near major sources such as roads, particularly in densely populated urban areas where strong local UFP emissions are associated with rapid changes over short distances^26^. This can obscure the details of UFP distributions that are critical for accurate exposure assessment.

To overcome these limitations, we present three key contributions in this study. First, we develop the first global maps of particle number concentration (PNC) at a 1 km spatial resolution, bridging the critical gap between local-scale land use regression models and coarse-resolution chemical transport models. Second, we introduce a machine learning framework that integrates limited ground measurements with diverse auxiliary data to predict PNC on a global scale, leveraging the XGBoost machine learning (ML) model for its capability to capture complex, non-linear relationships. Finally, we implement a statistically robust uncertainty quantification approach using conformal prediction, which provides reliable coverage intervals without depending on the assumption of normal data distribution.

Note the currently highest resolution global population data are also available on a 1 km grid, implying our health assessment studies can be performed by combining these datasets. Our methodology leverages ground station measurements worldwide and incorporates diverse auxiliary information, including the degree of urbanisation, built-up volume, anthropogenic emissions and combustion-related pollution concentrations. The XGBoost regression model predicts annual average PNC at 1 km spatial resolution over land, while the conformal prediction framework provides statistically robust 95% coverage intervals without prior assumptions of the data distribution. Additionally, we implement SHAP (SHapley Additive exPlanations) to investigate how the model reaches its predictions across different locations and environmental characteristics.

To assess the reliability of our predictions, we evaluated the model’s performance using multiple validation strategies. The XGBoost model achieved an R^2^ of >0.90 on the test dataset. Spatial and temporal cross-validation further demonstrated the applicability of the model, with R^2^ values between 0.77 and 0.87, respectively.

Our approach provides high-resolution PNC and UFP estimates that can support exposure assessment studies, particularly in regions lacking ground-based measurements. Section 2 describes the data sources and machine learning methodology, Section 3 presents the global PNC distribution patterns and model validation results, and Section 4 discusses the implications for air quality management and public health research.

## Methods

In the first part, we discuss the data sources and the data fusion methodology we utilised to standardise and homogenise them, from which the training and inference datasets were created. In the second part, we provide the specifics of our modelling approach, describing the training procedures and model performance evaluation using relevant metrics.

### Particle number concentrations

In acquiring the target variable for the ML model, we employed an approach fusing data from distinct sources. Initially, we accessed the EBAS database, which serves as the official outlet for the European Monitoring and Evaluation Programme (EMEP) and is hosted and operated by the Norwegian Institute for Air Research (NILU)^28^. We queried the database using the *pyebas* (https://github.com/defve1988/pyebas) Python API to retrieve all the available data for particle size distribution, PSD (*particle*_*number*_*size*_*distribution* component) and, particle number concentration, PNCs (*particle*_*number*_*concentration* component) for the years 2000–2020. Subsequently, we converted PSD data to PNC, by summing over the size distribution for each time step, and calculated the yearly average.

The data was retrieved in NetCDF format and for the *particle_number_size_distribution* and *particle_number_concentration* variables were used for PSD and PNC, respectively. The data entries were filtered with respect to the reported flag IDs; only entries with flag ID 000 (Valid measurement) and flag ID 100 (Checked by data originator. Valid measurement, overrides any invalid flags) (https://projects.nilu.no/ccc/flags/) were used. To ensure adequate representation of extended-term means, we excluded years with less than 150 unique days with available data. Furthermore, for the PSD data, the logarithmic diameter sizes were converted as follows:

For discrete diameter values ${\{{D}_{k}\}}_{k=0}^{N-1}$:

1 $$\begin{array}{lcc}\,{\rm{Bin\; borders}}\,{\{{b}_{i}\}}_{i=0}^{N}: &  & \\  & {b}_{i}=\left\{\begin{array}{ll}1{0}^{\left(1.5\frac{\log {D}_{0}}{\log 10}-0.5\frac{\log {D}_{1}}{\log 10}\right)}, & i=0\\ 1{0}^{\frac{1}{2}\left(\frac{\log {D}_{i-1}}{\log 10}+\frac{\log {D}_{i}}{\log 10}\right)}, & 1\le i\le N-1\\ 1{0}^{\left(1.5\frac{\log {D}_{N-1}}{\log 10}-0.5\frac{\log {D}_{N-2}}{\log 10}\right)}, & i=N\end{array}\right. & \end{array}$$

2 $$\begin{array}{ccc}\,{\rm{Logarithmic\; bin\; sizes}}\,{\{{\Delta }_{j}\}}_{j=0}^{N-1}: &  & \\  & {\Delta }_{j}=\frac{\log ({b}_{j+1}/{b}_{j})}{\log 10}={\log }_{10}\left(\frac{{b}_{j+1}}{{b}_{j}}\right) & \end{array}$$

Similarly, we retrieved PNC data from the Global Monitoring Laboratory (GML, https://gml.noaa.gov) of the National Oceanic and Atmospheric Administration (NOAA) agency for the same time period. The database was queried for the aerosol category and download the corresponding *particle*_*n*_*umber*_*c*_*oncentration* datasets for the available stations in .nas format. The *conc* variable was used and the data entries were filtered according to the reported *numflag* entry (only entries with flag = 0 were used), and stations with data in less than 150 days in each year were omitted.

**Fig. 1:**
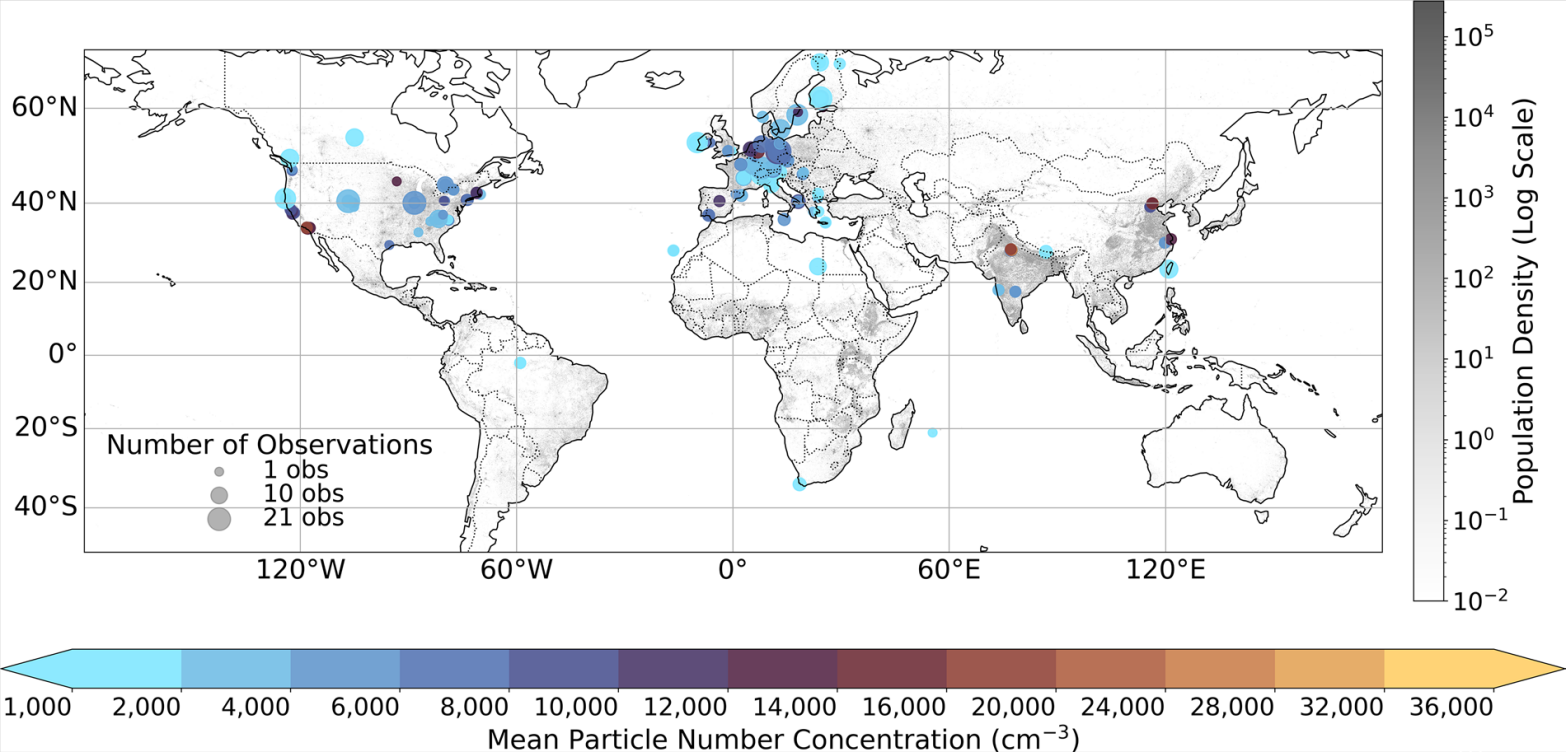
Geographical distribution of measurement locations in the dataset. Circle sizes indicate the number of observational datasets from each location (ranging from 1 to 21), while colours represent the mean particle number concentration (PNC) in cm^−3^ at each location. The background greyscale map shows global human population density on a logarithmic scale, providing context for the spatial relationship between measurements and population centres.

In addition, we conducted an extensive literature review to supplement the ground station data with information derived from published scientific articles presenting yearly PNC averages worldwide^21,25,29^. This literature review aimed to supplement the comprehensiveness of our dataset, by including measurements from diverse geographical locations and monitoring networks. Table 1 presents a summary of the unique entries, locations and the resulting yearly observations we were able to generate from each source. Figure 1 shows the geographical distribution of measurement locations in our dataset. Each location is represented by a circle, where both the circle’s diameter and colour indicate the mean PNC averaged over all available years at that site.

**Table 1:** Summary table of the PNC and PSD data used for training the machine learning model in this study.

Type	Unique locations	Yearly observations	Unique entries	Reference
PSD	55	403	137,421	EBAS^28,85–103^
PNC	37	351	2,585,052	EBAS^28,104–211^
PNC	6	17	17	GML - NOAA^212^
PNC	20	20	20	Kohl *et al*.^25^
PSD	17	34	34	Kohl *et al*.^25^
PNC	38	45	45	Saha *et al*.^21^
PNC	8	13	13	Aalto *et at*.^29^
**Total**	155	836		

Unique entries refers to individual measurements, whereas yearly observations refers to the final yearly aggregated data points used in training/evaluation of the ML models.

The 836 annual PNC observations listed in Table 1 originate from 155 distinct sites and 2.6 million individual sub-daily measurements acquired with condensation particle counters (CPC), mobility particle size spectrometers (SMPS/MPSS), and optical particle counters (OPC). Instrument classes, size-bin definitions, and temporal resolution differed across networks: EBAS and NOAA-GML stations typically report PSDs at 10-minute to 1-hour resolution, whereas literature compilations often provide daily or campaign-mean values. To harmonize these data, all records were re-screened for network quality flags, retaining only values flagged as valid or verified; sub-daily data were aggregated to daily means and subsequently to annual means provided that at least 150 unique days per year were available, a threshold commonly adopted in long-term aerosol climatologies to balance representativeness and data yield^30^. For data from stand-alone CPCs reporting only total number concentration, no size harmonization was necessary since CPC lower cut-offs lie within 3–10 nm, including the full ultrafine particle range relevant for this work.

#### Global human settlement layer

The Global Human Settlement Layer (GHSL) by the European Commission offers open and freely accessible data and tools for evaluating human presence and activities. In this work, the global built-up volume (GHS-BUILT-V) dataset was employed^31,32^, which includes both residential and non-residential buildings, encompassing industrial and commercial complexes. Additionally, datasets such as the degree of urbanization (GHS-SMOD)^32,33^ and human settlement (GHS-POP)^34,35^ were used. The GHSL datasets were employed to provide insight into anthropogenic activities and industrialization indicators, such as instances where a high built-up volume coincides with a low population density, potentially signalling the presence of industrial zones or other high-emission activities.

The datasets were retrieved in GeoTIFF format (WSG84 projection) from Copernicus and no temporal or spatial interpolations were conducted, and the closest year available for each of the datasets was utilized, as these variables do not change much over time. Given the strong linkage between emissions and human activity, these datasets can serve as proxies for pollution emissions.

#### Global NO_2_ and PM_2.5_

Two global datasets of NO_2_ and PM_2.5_ were incorporated into the feature set to determine the yearly average concentration of these air pollutants^36,37^. These datasets provide the yearly average concentrations of NO_2_ and PM_2.5_ for each grid cell. The *NO*_2_ datasets were retrieved in GeoTIFF format, whereas the *PM*_2.5_ datasets in NetCDF format. In both datasets, each yearly average was downloaded as a separate dataset. We note that these datasets were specifically generated for epidemiological and health burden studies, similar to the scope of this work.

We opted to include both ambient concentrations of *NO*_*x*_ and *PM*_2.5_ as well as emission inventories as input features in the model to capture the multiple processes influencing particle number concentrations at high resolution. Ambient concentrations reflect not only direct emissions, but also the effects of atmospheric dispersion, chemical transformation, remove processes and regional background levels, which emissions data do not capture. Moreover, background pollutant concentrations provide essential information on baseline exposures and long-range transport, especially between urban and peri-urban areas^22,38^.

The base spatial grid utilized throughout this study was constructed on the orthogonal latitude-longitude grid of the NO_2_ dataset. Furthermore, constrained by the latitude range of the PM_2.5_ dataset, this study spans latitudes ranging from 55^º^S to 68^º^N degrees.

#### Emissions

The gridded distributions of global anthropogenic emissions from the Copernicus Atmosphere Monitoring Service (CAMS) were utilized to obtain combustion-related emissions data^39^. The dataset comprises modified Copernicus Atmosphere Monitoring Service Information for the year 2023, retrieved from the Copernicus Atmosphere Data Store. The global emission inventory from CAMS was utilized to derive proxies to estimate PNCs and consider anthropogenic contributions, especially from combustion sources, by including yearly average emissions of black carbon (BC), carbon monoxide (CO), carbon dioxide (CO_2_) and nitrogen oxides (NO_*x*_).

The *cams-global-emission-inventories* dataset was queried using the cdsapi in Python and a separate NetCDF file was retrieved for each year/variable combination, for the *black*_*carbon*, *carbon*_*monoxide*, *carbon*_*dioxide* and *nitrogen*_*oxides* species. The datasets contain both individual sector emissions and the cumulative sum, with the total variable selected for each species (*sum* variable). Emphasis was placed on emissions over land, thus, grid cells classified as 100% “open sea” were excluded. Only emissions resulting from combustion processes were considered for this study.

The yearly averages per grid cell were calculated using the *resample* method of the Python *xarray* library. Spatial interpolations were performed to redistribute the emissions in each grid cell with respect to population density and built-up density, as described below.

#### Temperature

The fifth-generation ECMWF reanalysis for global climate and weather, ERA5, served as the source for the temperature feature in our analysis, which may be viewed as a proxy for meteorological conditions. Specifically, the 2m temperature (*t2m*) variable was obtained format from the Copernicus Climate Change Service (C3S) Climate Data Store (CDS)^40^. Temperature was included as a parameter due to its potential to influence and reflect atmospheric processes. Temperature can also affect UFP formation and growth through photochemical oxidation of volatile organic compounds (VOCs) and nitrogen oxides (NO_*x*_), as well as condensation and evaporation of semi-volatile reaction products^41,42^. The dataset contains modified Copernicus Climate Change Service information (2023), retrieved from the Copernicus Atmosphere Data Store. Yearly averages for each grid cell were computed using the *resample* method of the *xarray* library in Python 3.11, and no spatial interpolations were applied during this process.

#### Boundary layer height

The Boundary Layer Height (BLH) was also incorporated from the ERA5 reanalysis dataset. BLH directly relates to the vertical mixing and dilution of particles in the lower atmosphere, thus, by incorporating BLH data we aim to account for the influence of atmospheric stability on surface particle concentrations. Shallow boundary layers, typically occurring during nighttime or winter conditions, lead to particle accumulation near the surface, while deeper boundary layers are associated with enhanced vertical mixing and dilution^43^. The ERA5 dataset was also used for this variable; the CDS datastore was queried for the *blh* variable using the cdsapi, with separate NetCDF files downloaded for each year.

#### Precipitation

Precipitation is a relevant meteorological parameter for PNC prediction, as it plays an important role in particle removal through wet deposition processes. We have incorporated the total precipitation for each grid cell from the ERA5 reanalysis dataset (*tp* variable, obtained using the cdsapi in NetCDF format), which accounts for one of the primary removal pathways of atmospheric particles. Wet deposition is especially important in regions with frequent precipitation events, where particle removal can substantially influence the annual average concentration that our model aims to predict^44,45^.

#### Road network

The Global Roads Open Access Data Set v1 (gROADSv1) is a comprehensive global road network database that incorporates the major roads and highways worldwide. We included this dataset as road traffic represents one of the primary sources of ultrafine particle emissions in urban environments. The dataset provides detailed spatial information about traffic networks on a global scale, by including roads and highways as line-shapes in shapefile format. To convert to a gridded dataset, we calculated the number of roads intersecting every cell of the global grid, as a proxy for capturing the traffic-related particle emissions. This kind of information is important to this study since vehicle exhaust emissions have been shown to create strong spatial gradients in particle number concentrations, with elevated levels typically observed near major roadways and traffic corridors^46^.

#### Population

The global population dataset from WorldPop (www.worldpop.org) was incorporated into our analysis^47^, which provides population counts on a global scale. The data were obtained directly from the organization’s website, without any temporal or spatial manipulation.

Data spanning the years 2000 to 2020 were retrieved to ensure a comprehensive temporal coverage for our analysis. The WorldPop population counts dataset serves as a fundamental resource in our study, offering insights into the spatial distribution of human populations across diverse regions worldwide.

#### Data homogenization

The NO_2_ dataset at 0.01^°^ grid resolution, roughly 1 km at the equator and a decreasing longitude extent towards the poles (about 0.5 km at 60^°^ latitude), served as the baseline for establishing a uniform gridded dataset. This dataset functioned as the reference point for aligning the spatial resolution of other datasets, ensuring consistency throughout the training and inference processes. To integrate land use data into the uniform dataset, the 100 grid points within each 1km grid cell were identified. For each land use class, the percentage coverage was extracted, resulting in seven features.

Datasets sharing the same spatial resolution as that of NO_2_, such as the PM_2.5_ and the GHSL data, were seamlessly integrated into the uniform gridded dataset, ensuring the coherence of the datasets without introducing discrepancies.

To address the spatial resolution disparity between the emissions dataset (10km grid) and other datasets (1km grid), a redistribution process was executed. This downscaling process maintained the total emissions within each 10 km grid cell (*Em*_10*km*_) while redistributing them to a 1km resolution (*Em*_1*km*_). Downscaling was achieved by linearly weighting emissions based on population and built-up volume, ensuring harmonisation with other datasets, following Kohl *et al*.^25^, as follows:

3 $$E{m}_{1km}=E{m}_{10km}\times \frac{(Po{p}_{1km}/Po{p}_{10km}+B{V}_{1km}/B{V}_{10km})}{2}$$

where, *Pop*_1*km*_ and *BV*_1*km*_ is the population density and built-up volume in the 1km grid cell, respectively, and *Pop*_10*km*_ and *BV*_10*km*_ the total population density and build-up volume in the 10 km grid cell.

Finally, Table 2 provides a list of the feature set employed in this study, as well as the temporal and spatial resolution of the datasets. By implementing the aforementioned procedures, we arrived at a dataset comprised of 836 examples of PNC concentrations characterised by a set of 14 features, which we used for the training and evaluation procedures.

**Table 2:** The input feature set used to train the ML models and during the inference procedures.

Category	Feature Name	Resolution	Reference
Human Activity	Population	1 km - Yearly	^47^
	Build-up volume	1 km - 5 Years	^31^
	Degree of urbanisation	1 km - 5 Years	^33^
	Human settlement	1 km - 5 Years	^34^
	Road network	Line geometry - Static	^46^
Air Quality	NO_2_ concentration	1 km - Yearly	^36^
	PM_2.5_ concentration	1 km - Yearly	^37^
Emissions	Black carbon	10 km - Monthly	^39^
	Carbon dioxide	10 km - Monthly	^39^
	Carbon monoxide	10 km - Monthly	^39^
	Nitrogen oxides	10 km - Monthly	^39^
Meteorological	Temperature	25 km - Hourly	^40^
	Boundary layer height	25 km - Hourly	^40^
	Precipitation	25 km - Hourly	^40^

#### UFP estimation from PNC

**Fig. 2:**
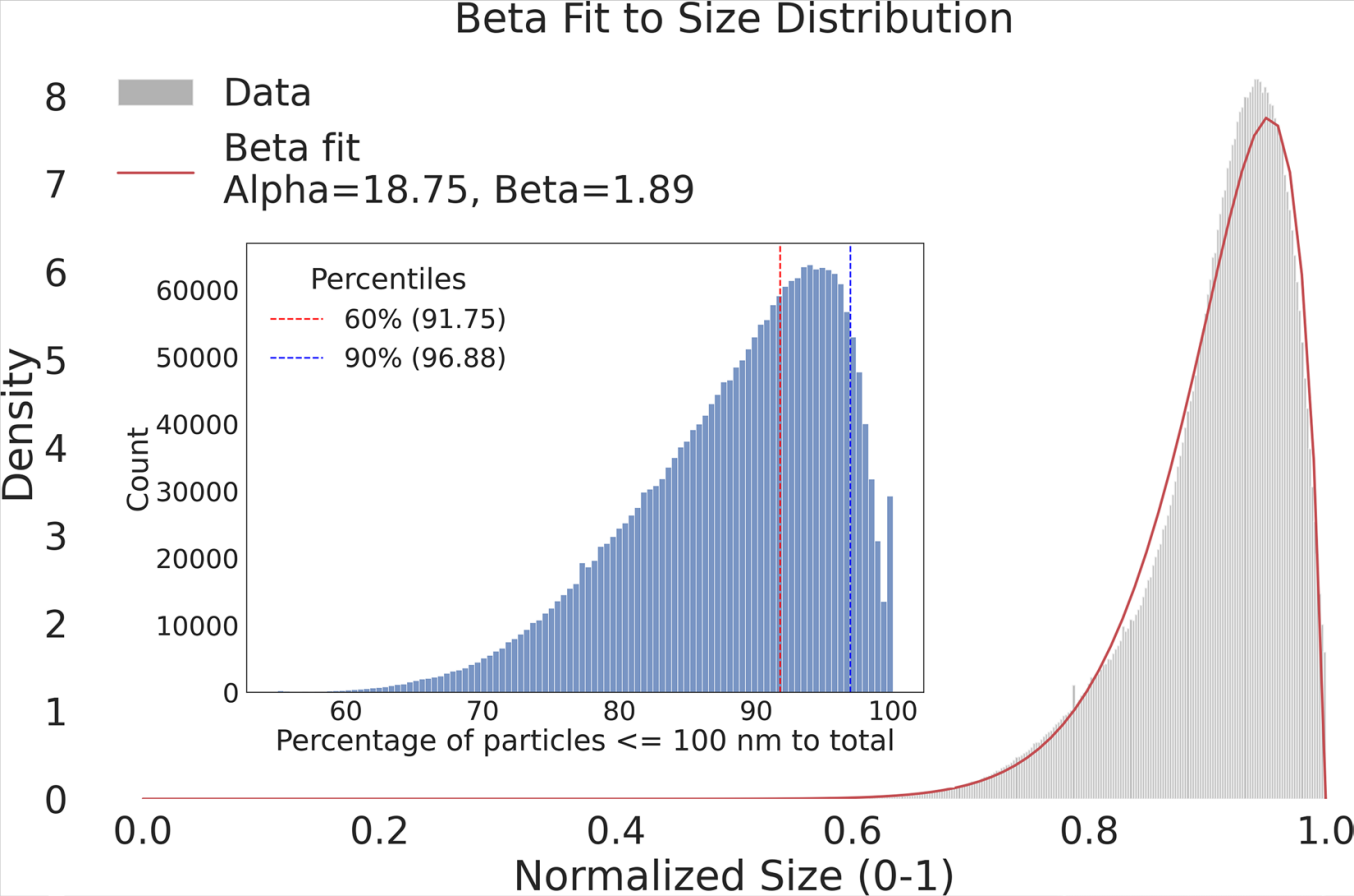
Beta distribution fit to the normalized UFP fractions relative to total PNC. The main plot shows the fitted Beta distribution, capturing the variability in UFP fractions. The inset displays the histogram of the percentage of particles under 100 nm with respect to the total PNC, with vertical dashed lines indicating the 60% (red) and 90% (blue) percentiles.

To estimate UFP concentrations from PNC measurements, we analysed particle size distribution (PSD) data from the EBAS database. Figure 2 shows the distribution of the UFP fraction (particles <100 nm) relative to total PNC across all available measurements. The analysis reveals that UFPs dominate the total particle count in most locations, with a mean contribution of 91%. This aligns with studies in traffic-dominated urban areas where vehicular emissions (a primary source of UFPs) account for >90% of PNC^12^. However, regional studies highlight variability in UFP/PNC ratios due to differences in emission sources and atmospheric processes^48^. In urban and roadside environments, UFP fractions >90% are typical due to traffic emissions, consistent with our mean estimates. In industrial and coastal areas, UFP fractions can be lower (70-85%) as particle emissions are dominated by industrial coarse-mode particles (e.g. metal processing) or marine aerosols (e.g. sea spray and ship emissions)^49^. Furthermore, in rural and suburban regions with strong new particle formation (NPF), UFP fractions are often higher (>95%)^50^.

To quantify uncertainties, we fitted a Beta distribution (shape parameters *α* = 18.75, *β* = 1.89) to the normalised UFP fractions (Fig. 2). The derived mean (0.9082) and 95% coverage interval ([0.7866, 1.0299]) reflect variability in our dataset, they are consistent with the ranges reported in the literature for urban and highly populated regions, which is the primary focus of this study. Applications in industrial or coastal regions may require localised adjustments.

### Methodology

#### XGBoost

In this study, we apply the Extreme Gradient Boosting (XGBoost) algorithm to estimate PNCs and UFP concentrations. The XGBoost algorithm was chosen for its computational efficiency, scalability, and recognized track record in performance and flexibility. It utilizes an ensemble tree-based learning scheme, which can effectively handle mixed data types, resist outliers, and model complex, non-linear relationships without overfitting^51–53^.

The XGBoost model combines predictions from multiple decision trees, where each subsequent tree learns to correct the errors of its predecessors. This makes it particularly effective at capturing complex relationships between environmental factors and particle concentrations. The mathematical framework consists of three key components:

**Prediction framework**

The model is built stage-wise, with predictions given by:

4 $${\widehat{y}}_{i}=\mathop{\sum }\limits_{k=1}^{K}\,{f}_{k}({{\bf{x}}}_{i}),\quad {f}_{k}\in {\mathcal{F}}$$

where ${\widehat{y}}_{i}$ represents the predicted UFP concentration, *K* is the number of trees, and each tree *f*_*k*_ maps environmental inputs **x**_*i*_ to concentration estimates.

**Loss Function**

The objective function balances model fit against complexity:

5 $${\mathcal{L}}(\theta )=\mathop{\sum }\limits_{i=1}^{n}l({y}_{i},{\widehat{y}}_{i})+\mathop{\sum }\limits_{k=1}^{K}\Omega ({f}_{k})$$

where *n* is the number of examples in the dataset, *l* measures prediction accuracy using mean squared error, and *Ω* controls model complexity.

**Regularization**

To prevent overfitting, the regularization term is defined as:

6 $$\Omega (f)=\gamma T+\frac{1}{2}\lambda \mathop{\sum }\limits_{j=1}^{T}{w}_{j}^{2}$$

where *T* represents leaf count, *w*_*j*_ are leaf weights (optimal prediction scores) calculated as ${w}_{j}=-\frac{{G}_{j}}{{H}_{j}+\lambda }$, with *G*_*j*_ and *H*_*j*_ being the sum of gradients and Hessians respectively for instances in leaf *j*, and *γ* and *λ* control the regularization strength^51^.

#### Training and evaluation

To determine the optimal set of parameters for the model, we divided the dataset into training and test sets, with a 90/10 split. This ensured that the test portion of the data was not utilized during the hyperparameter tuning process. Following this, the remaining data was further subdivided into a training and validation set, following a 90/10 split. We performed an exhaustive grid search in parameter space and assessed the performance of each model using the validation set. The parameter space explored in this study included the following ranges: the number of estimators varied between 30 and 250, while the number of parallel trees ranged from 1 to 10. The maximum depth of the trees was set between 3 and 15, and the learning rate spanned from 0.01 to 0.5. Additionally, the subsample ratio of the training instances was tested between 0.3 and 1, and the subsample ratio of columns used for constructing each tree also ranged from 0.3 to 1.

Once the optimal set of parameters was determined, we employed multiple validation strategies to thoroughly assess model performance and generalizability:

- **K-fold cross validation**. The training dataset was randomly partitioned into K folds (10-fold in this case), where 90% of the data was used for training and 10% for evaluating the performance relative to unseen data.
- **Spatial Leave-One-Out Cross Validation (LOOCV)**. Using the complete dataset to ensure comprehensive spatial coverage, the data was partitioned with respect to the location of the ground stations. In each iteration, the data from one ground station was left out to be used as a validation set and the model was trained on the rest of the data, to assess generalizability to unseen locations.
- **Temporal LOOCV**. Similarly, to evaluate temporal generalizability, we used the complete dataset partitioned by year. In each of the twenty cross-validation iterations (2000–2020), one year was left out and the model was trained using the rest of the data.

Finally, we evaluated the model’s performance on the held-out test set, which remained completely unused during both hyperparameter optimization and cross-validation procedures. This provided an unbiased assessment of the model’s effectiveness using standard metrics including Mean Absolute Error (MAE), Mean Squared Error (MSE) and the coefficient of determination (R^2^). This iterative process allowed for the evaluation of the model’s performance across multiple validation sets, which enabled us to quantify the spatial and temporal generalizability of the model.

#### Conformal prediction with XGBoost

To assess the prediction performance of the model, we used the conformal prediction statistical framework to estimate the uncertainties of the model results. Conformal prediction provides a mechanism to generate statistically valid coverage intervals associated with the results of traditional ML models. Coverage intervals in this framework are distribution-agnostic, unlike similar methods like Natural Gradient Boosting and Gaussian processes, which assume data is normally distributed, an assumption that often fails in real world datasets^54^. We used the Model Agnostic Prediction Interval Estimator (MAPIE) library in Python 3.11 to implement conformal predictions with the XGBoost Regressor implementation of the *xgboost* library.

In general, conformal predictions operate by training the base model and calculating the coverage intervals using a holdout set of data. In this study, due to the limited number of long-term particle concentration data available, we used the Jackknife+ after Bootstrap method to enhance the robustness of our coverage intervals. This method involves the following steps:

- **Bootstrap Resampling**. In the first step of the process, the training dataset is resampled multiple times (in this case 20), to create several bootstrap samples. The XGBoost regression model is trained separately on each of these samples.
- **Leave-One-Out predictions**. For each bootstrap sample, leave-one-out (LOO) predictions are made, where each instance in the sample is left out once during the prediction process.
- **Nonconformity scores**. The nonconformity of each prediction is assessed by comparing the LOO predictions to the actual values in terms of the mean-squared error. These scores measure how well the predictions conform to the observed data.
- **Interval calculation**. The distribution of the nonconformity scores across all boostrap samples is used to determine the bounds of the prediction intervals for new data points, based on the desired coverage intervals (in this case *α* = 0.05, or 95% coverage interval).

The jackknife+ after bootstrap approach guarantees a coverage level (the amount of observed data that lie within the predicted coverage intervals of the model) higher than 1–2*α* for a target coverage level of 1-*α*, without any a priori assumption on the distribution of the data, where *α* is the confidence interval^55,56^.

#### Explainability

To gain insights into the underlying fundamental operation of the ML model, we utilised the SHAP (SHapley Additive exPlanations) method. Shapley values, based on a commonly used approach from cooperative game theory, assess the individual contribution of each input feature to a specific prediction, which allows us to identify and quantify the features that contribute the most to the model’s output^57^. The core concept behind SHAP involves comparing the model prediction for a single data point to what it would have predicted under various hypothetical scenarios, where certain features are “masked out”. By aggregating these individual feature contributions, SHAP assigns an attribution value to each feature, indicating its impact on the final prediction^58^.

Mathematically, the model is retrained on all feature subsets *S* ⊆ *F*, where *F* is the entire feature set. The importance value is assigned to each feature that represents the effect on the model output including that feature. To compute this effect, two models are trained, one with the feature present (*f*_*S*∪{*i*}_) and one with the feature withheld (*f*_*S*_). The predictions from the two models are then compared for each input *f*_*S*∪{*i*}_(*x*_*S*∪{*i*}_) − *f*_*S*_(*x*_*S*_), where *x*_*S*_ represents the values of the input features in the set *S*. As the effect of removing a feature is dependent on other features in the model, the preceding differences are computed for all permutations of the subset *S* ⊆ *F*\{*i*}. The Shapley values are subsequently computed as feature attributions and are a weighted average of all possible differences^58,59^:

7 $${\phi }_{i}=\sum _{S\subseteq F\backslash \{i\}}\frac{| S| !(| F| -| S| -1)!}{| F| !}\,[{f}_{S\cup \{i\}}({x}_{S\cup \{i\}})-{f}_{S}({x}_{S})].$$

A positive SHAP value suggests the feature improves the model prediction, while a negative value indicates the feature operates in the opposite direction. The magnitude of the value reflects the strength of the influence^60^.

We utilized the SHAP library in Python and the TreeExplainer method to generate beeswarm visualisations^58^. These plots served to elucidate the feature attributions in the model and their influence on individual predictions, respectively. SHAP is a model-agnostic framework that computes feature attributions, explaining how each feature contributes to a specific prediction. In this case, the TreeExplainer method leverages tree-based ML models to calculate these attributions. It does so by creating a set of decision trees that mimic the behaviour of the original model. By analysing how each feature splits the data within these trees, the explainer can determine the contribution of each feature to the final prediction.

## Data Record

The dataset consists of global maps depicting particle number concentration (PNC) for each year spanning 2010 to 2019 at a spatial resolution of 1 km. The full dataset is freely accessible in the Zenodo repository under a Creative Commons Attribution 4.0 International (CC-BY 4.0) license at 10.5281/zenodo.14832351^61^ It is distributed as ten separate NetCDF files, each corresponding to one calendar year within the covered period.

Each NetCDF file contains annual mean PNC values stored in a variable labeled PNC, 95% coverage intervals indicating uncertainty in a variable labeled CI, and estimated ultrafine particle (UFP) concentrations in a variable labeled UFP. All these variables are defined on a uniform 1 km latitude-longitude grid covering the global land surface. The particle concentrations are expressed in units of particles per cubic centimeter (cm^−3^). Metadata embedded in each file describes the variable attributes, coordinate system, and provenance details to facilitate proper interpretation and reuse.

The naming convention for the files follows the pattern YYYY.nc, where YYYY is the year designation from 2010 through 2019. The NetCDF format ensures compatibility with a wide range of geospatial and scientific computing software tools. Spatial coordinates correspond to standard geographic latitude and longitude dimensions. No additional processing is required to access or utilize the data beyond typical NetCDF file handling.

**Fig. 3:**
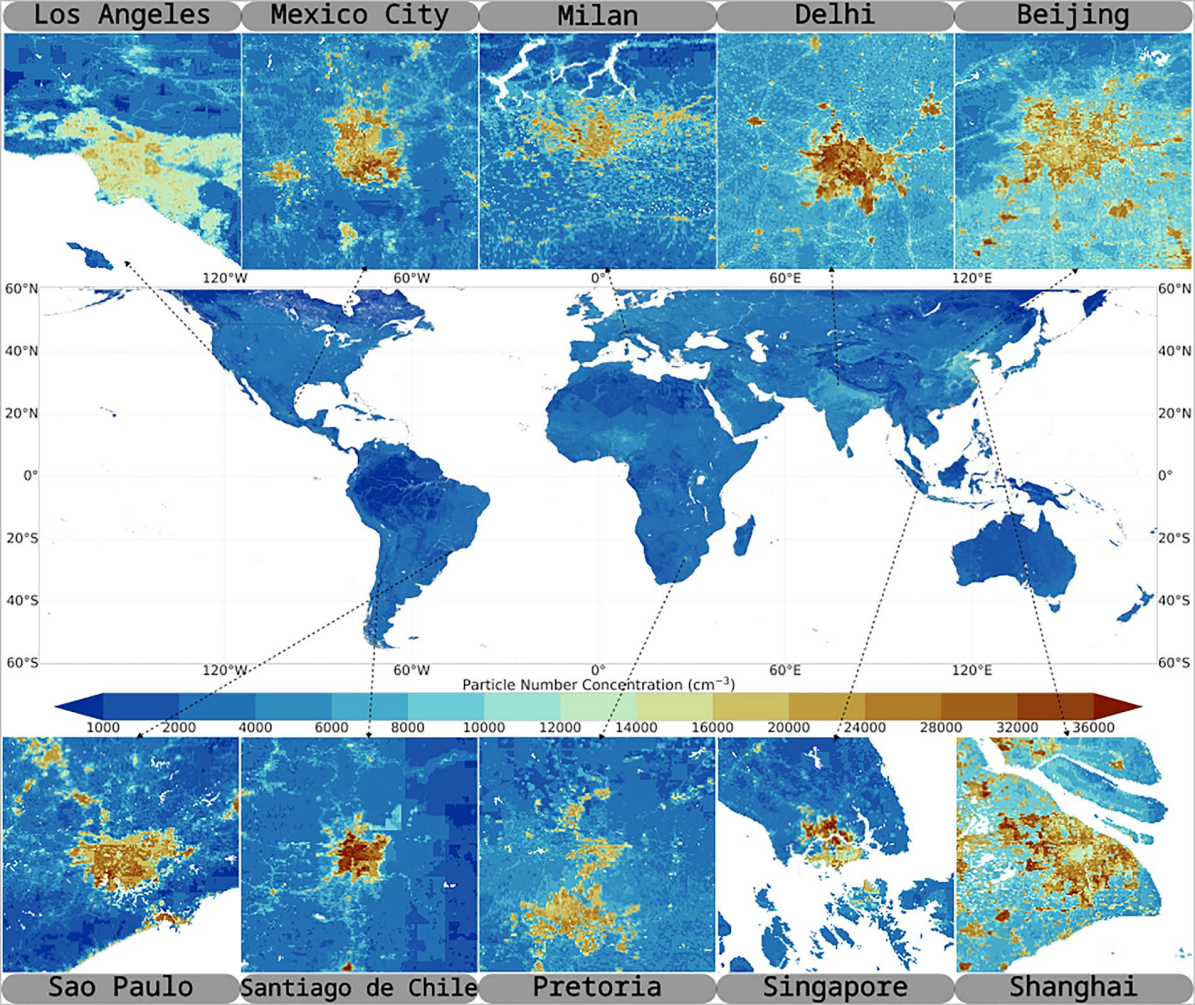
Global distribution of particle number concentration (PNC) at 1 km resolution. Center: Global map of predicted PNC values (cm^−3^). Top and bottom panels show zoomed-in views of selected cities around the world, highlighting the fine-scale spatial variations in PNC and their relationship with urban structure and emission sources.

Figure 3 shows an example illustration of the dataset structure for the year 2015, intended solely as a visualization of the data layout on the global grid.

## Technical Validation

### Model performance

**Fig. 4:**
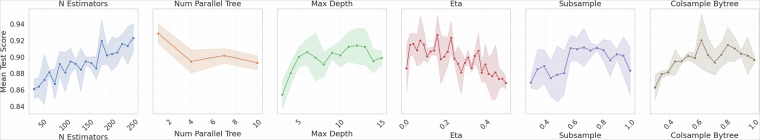
Sensitivity analysis results obtained by performing an exhaustive search in parameter space using the grid search method.

We evaluated the performance of the XGBoost model using multiple validation strategies to ensure robust predictions of global PNC distributions. Through an exhaustive grid search, we identified the optimal hyperparameters for the model as follows: the number of estimators was set to 250, with a single parallel tree. The maximum tree depth was determined to be 10, and the learning rate (*η*) was 0.03. Additionally, the subsample ratio of training instances and the subsample ratio of columns were both set to 0.75. Figure 4 presents a sensitivity test, obtained from the results of the fitted models during the exhaustive search in parameter space for tuning the hyperparameters of the XGBoost model (evaluated on the held out portion of the data in each iteration).

**Fig. 5:**
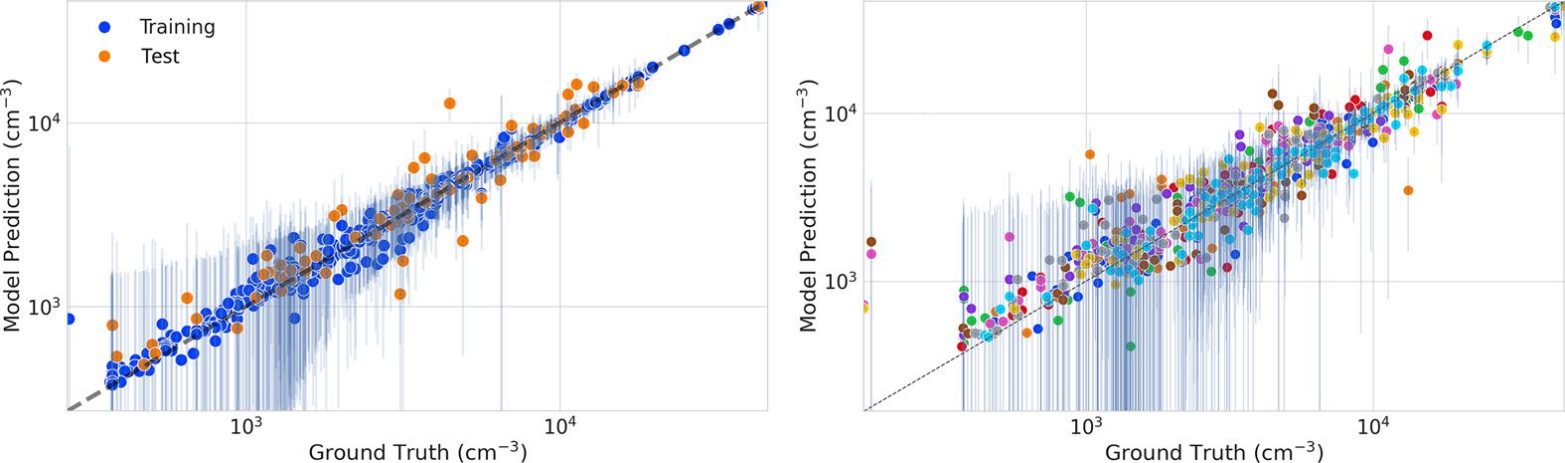
Left: Predicted versus observed PNC values for the training (90%) and test (10%) datasets. Right: Predicted versus observed PNC values from 10-fold cross-validation, showing only out-of-fold (held-out) predictions for each fold.

Using these parameters, Fig. 5 demonstrates the model’s predictive capabilities. The traditional train-test split evaluation yields an R^2^ of 0.90 and a Mean Absolute Error of 1336 cm^−3^, while the 10-fold cross-validation shows slightly better performance with an R^2^ of 0.91 and MAE of 1025 cm^−3^.

**Fig. 6:**
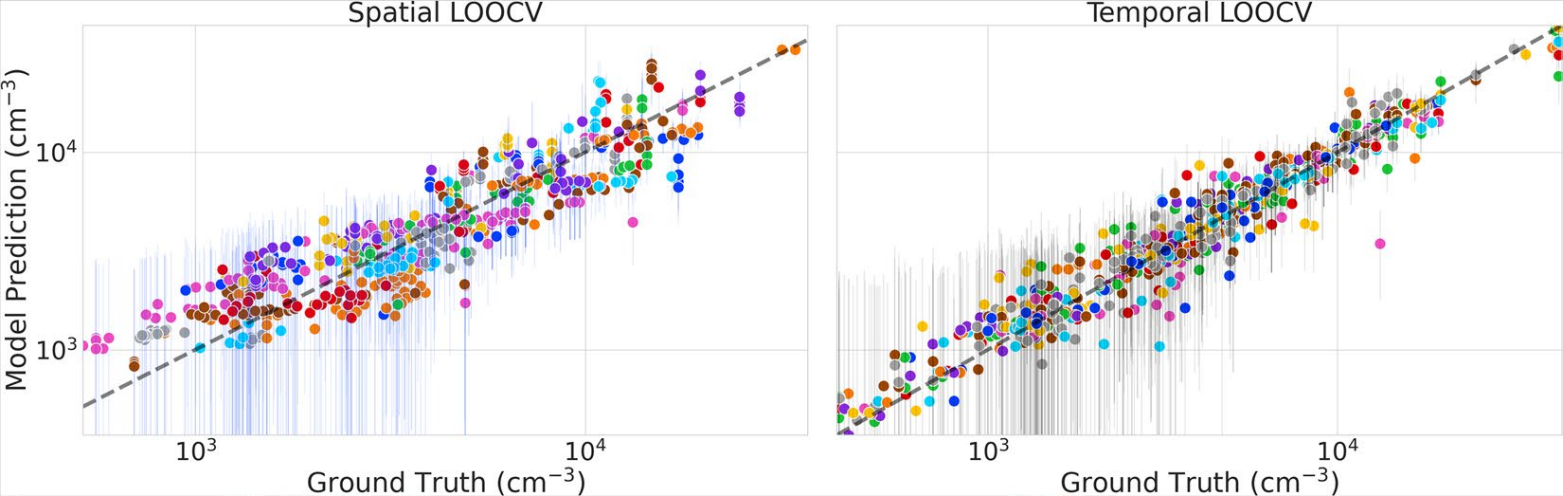
Model performance under different cross-validation schemes. Left: Predicted versus observed PNC values from spatial Leave-One-Out Cross-Validation, where measurement stations are held out. Right: Predicted versus observed PNC values from temporal Leave-One-Out Cross-Validation, where entire years are held out.

To assess the model’s ability to predict PNCs at new locations and times, we performed spatial and temporal Leave-One-Out Cross-Validation (LOOCV) (Fig. 6). The spatial LOOCV, where entire measurement stations are held out, achieves an R^2^ of 0.77 and MAE of 2,839 cm^−3^. This lower performance reflects the inherent challenge of spatial extrapolation to completely new locations, particularly given our limited number of measurement stations globally. The reduced spatial LOOCV performance of the model highlights a critical limitation in global PNC estimation, stemming from the uneven distribution of ground-based monitoring stations, especially in low- and middle-income regions such as Africa and South America. As shown in Fig. 1, the majority of ground station data currently originate from Europe and North America, resulting in data-sparse regions where extrapolation errors are more likely. This gap in monitoring coverage is a common challenge in global air pollution modelling, where data scarcity in developing regions introduces significant uncertainty in exposure assessments and model predictions^62^.

Sparse monitoring networks affect model generalizability and can lead to higher uncertainty and reduced predictive accuracy in regions without robust ground validation. Satellite-based approaches have made progress in addressing global gaps, but they also face limitations due to validation requirements and region-specific uncertainties^63^. It is therefore imperative to interpret predictions for data-poor regions with caution, as the model may not fully capture the local emission sources, meteorology, and atmospheric processes unique to these areas. To further quantify regional uncertainties, users are encouraged to refer to the model’s conformal prediction coverage intervals, which adaptively widen in areas with reduced training data density.

In contrast, the temporal LOOCV, where entire years are held out, demonstrates good performance with an R^2^ of 0.87 and MAE of 1,740 cm^−3^. This stronger temporal performance suggests that our model captures year-to-year variations relatively more effectively than spatial patterns, likely due to the more consistent nature of temporal processes governing PNC distributions. The better temporal generalisation also indicates that our chosen features effectively represent the dynamic processes controlling particle concentrations, even when predicting for unseen years.

The percentage errors remain relatively consistent across validation methods, ranging from 23% for the test set to 32% for spatial LOOCV (Table 3). Notably, the spatial LOOCV exhibits a prediction minimum around 1,000–1,500 cm^−3^, primarily due to the limited number of training stations in high-latitude regions, which were omitted due to being outside the latitudinal range of input variables.

**Table 3:** Model performance metrics for different validation strategies.

Method	MAE	MSE	R^2^	Perc. Error
10% Test set	1,336	2,644	0.90	23%
10-fold CV	1,025	1,541	0.91	24%
Spatial LOOCV	2,839	9,427	0.77	32%
Temporal LOOCV	1,740	6,912	0.87	26%

MAE and MSE are given in cm^−3^. The percentage error represents the mean relative error across all predictions. Results show performance for: traditional test set evaluation (10% of data), 10-fold cross-validation, spatial Leave-One-Out Cross-Validation (LOOCV) where individual stations are held out, and temporal LOOCV where entire years are held out.

These results represent the current state-of-the-art in global PNC prediction, considering the relative novelty of these measurements and the limited availability of long-term PNC monitoring data. Despite these limitations, the model demonstrates reliable extrapolation capabilities to new locations, providing a valuable tool for global PNC estimation.

The model’s performance varies significantly across different population density classifications, as shown in Table 4. In densely urbanised areas (>1,900 people/km^2^), where annual and global mean PNC values are highest at 14,992 cm^−3^, the absolute uncertainty is largest with a mean 95% coverage interval of 3715 ± 182 cm^−3^. Due to the high PNC values in these regions, this translates to a relatively small percentage error of 29 ± 2%. Suburban areas (250-800 people/km^2^) show intermediate values with a global mean PNC of 6,360 cm^−3^ and percentage error of 35 ± 3%. While rural areas (<250 people/km^2^) have the smallest absolute coverage interval (1852 ± 56 cm^−3^), they show the highest percentage error of 91 ± 3% related to their low mean PNC values (2,606 cm^−3^).

**Table 4:** Model predictions and uncertainty metrics across different population density classifications.

Classification	Population limit (km^−2^)	Mean PNC (cm^−3^)	Mean 95% CI	Mean Percentage Error
Rural	250	2,606	1,852 ± 56	91 ± 3
Suburban	800	6,360	2,165 ± 72	35 ± 3
Urban	1,900	14,992	3,715 ± 182	29 ± 2

Areas are classified as rural (<250 people/km^2^), suburban (250–800 people/km^2^), or urban (>800 people/km^2^). For each class, the table shows mean PNC values, 95% coverage intervals, and percentage errors (presented as mean  ± standard error).

This high percentage error in rural areas is not critical from an exposure assessment perspective, as these regions combine low population density with relatively low PNC values and minimal health outcomes. However, episodic pollution events, such as agricultural burning in Punjab, India, or Imperial Valley, California, can generate acute PNC spikes (e.g. more than 20,000 cm^−3^ during post-harvest seasons) linked to respiratory hospitalisations and developmental disorders in children^64,65^. Such events are underrepresented in long-term averages due to sparse monitoring and low baseline values, potentially biasing health studies that rely on annual means. Similarly, emerging rural pollution sources like biomass cook stoves in sub-Saharan Africa or mining activities in rural Mongolia may produce ultrafine particles (UFPs) that existing networks fail to capture, further complicating exposure-risk assessments^66,67^.

This variability in model performance is particularly important when considering the steady increase in the percentage of people living in suburban and urban environments. Based on WorldPop population counts, the proportion of the global population residing in these areas has been steadily increasing from  ~67% in 2000 to  ~73% in 2020, while the total population has increased from  ~6 billion to  ~8 billion people in the same time period^47^. This trend highlights the growing significance of accurately modelling air pollution exposure in suburban and urban regions, where both population density and PNC/UFP levels are comparatively high. The relatively low percentage error in urban areas enables reliable exposure assessments for a large and increasing share of the global population. Conversely, while rural areas exhibit higher percentage errors, their low population densities and lower PNC/UFP values mitigate the criticality of these uncertainties from an exposure assessment perspective. Nevertheless, additional measurement datasets in rural settings will be needed to improve the model performance across different environmental conditions.

Conformal prediction^55^ provides reliable uncertainty quantification even with the limited spatial coverage of the global measurement network. Unlike traditional methods that rely on distributional assumptions, conformal prediction offers distribution-free prediction intervals by leveraging the exchangeability of training and test data. While exchangeability may theoretically be violated in real-world settings-for example, due to gradual temporal trends in emissions or shifts in monitoring networks, such risks are mitigated in our analysis. First, the use of yearly-averaged data reduces sensitivity to short-term fluctuations, thereby weakening the impact of gradual temporal trends on exchangeability. Second, empirical validation demonstrated that uncertainty intervals maintained  ~95% coverage across held-out test sets spanning diverse regions and years, with no significant degradation in performance. Notably, intervals adapted to sparse measurement regimes by widening appropriately to reflect increased uncertainty, suggesting robustness to mild violations of exchangeability.

Although abrupt temporal shifts (e.g., rapid emission reductions following policy changes) could exacerbate exchangeability violations, such effects were not observed in our experiments. The framework’s practical robustness is further supported by strong temporal cross-validation results (R^2^ = 0.87), aligning with findings in^68^, where conformal prediction achieved near-target coverage despite mild exchangeability violations in environmental applications.

### Explainability

**Fig. 7:**
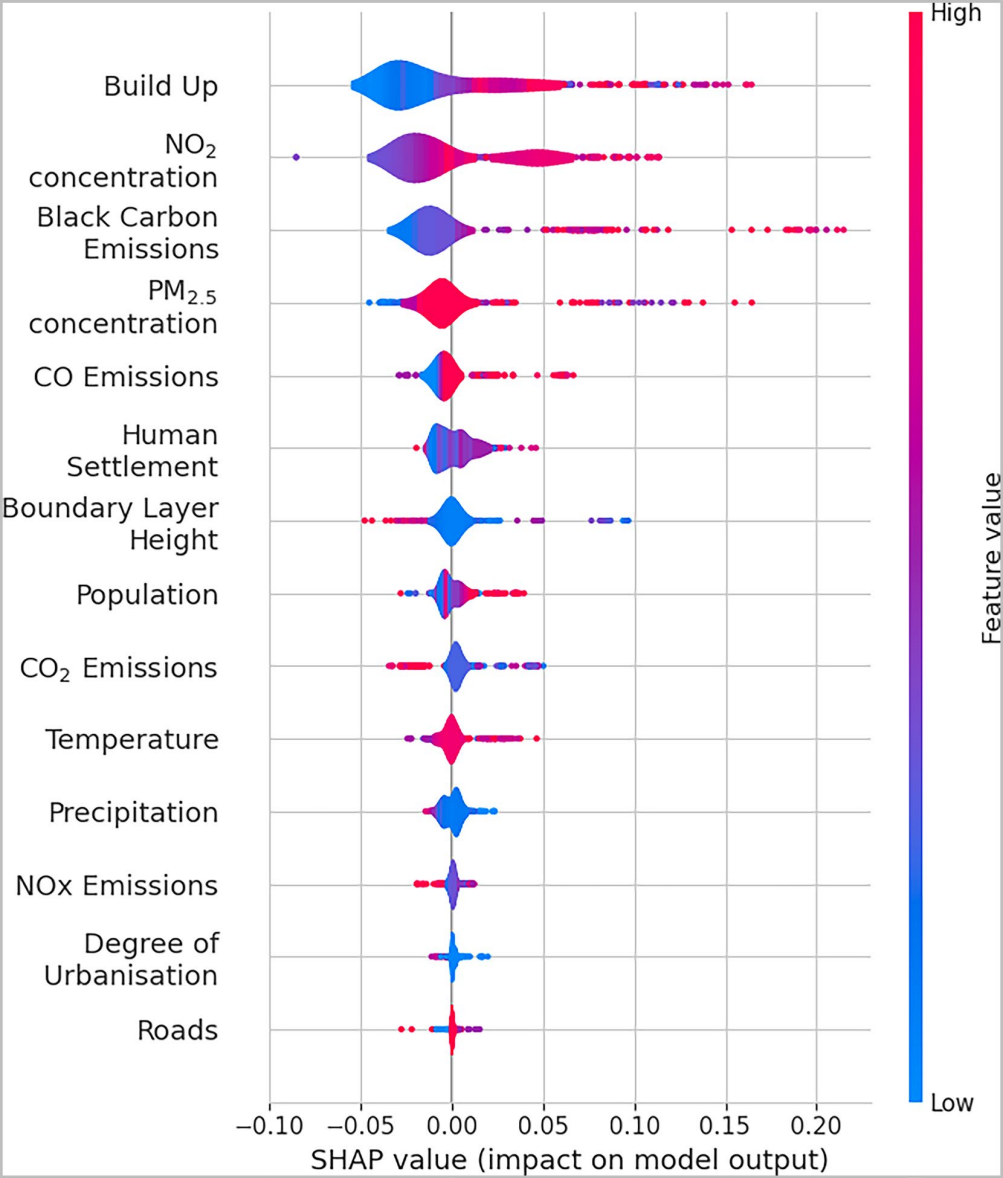
Feature importance analysis using SHAP values. The plot shows the impact of each feature on model predictions, where each point represents a single prediction. Features are ordered by their absolute SHAP values, with higher values indicating a stronger influence on PNC predictions. Colours represent the feature value (blue for low, red for high).

To understand how the features used in our machine learning model influence the model’s predictions, we employed the SHAP method. SHAP quantifies each feature’s contribution to individual predictions while accounting for feature interactions and providing insights into both the relative importance of features and how their values affect the model’s output. Figure 7 presents a beeswarm plot where features are ordered by their absolute impact on model predictions. Each point represents a single prediction, with its horizontal position showing the SHAP value (negative values indicate weakening of predictions, positive values enhance them) and its colour indicating the feature value (blue for low, red for high). The maximum SHAP value obtainable by a single feature would be one since the model output is scaled to the 0-1 range. For example, a SHAP value of 0.2 for built-up volume indicates that this feature can contribute up to approximately 9,000 cm^−3^ (20% of the maximum range of approximately 45,000 cm^−3^) to the final PNC prediction when its value is high.

Built-up volume emerges as the most important feature, followed by NO_2_ concentrations, black carbon emissions and PM_2.5_ concentrations, with maximum SHAP values up to approximately 0.2. The strong positive correlation between high built-up volume, NO_2_ and black carbon emissions with PNC aligns well with our understanding of particulate pollution in urban environments^20^.

Interestingly, PM_2.5_ shows a slight negative impact (up to around −0.025) even at high feature values, suggesting that processes governing particle number concentrations can differ from those controlling particle mass. PNCs are dominated by UFPs, which contribute significantly in terms of number but very little to mass concentration. In a study across multiple cities, de Jesus *et al*. have shown that PNC and PM_2.5_ measurements are not representative of each other^48^. The negative correlation can be attributed to differences in the formation processes and sources of the two pollutants. Apart from the differences in formation processes, PM_2.5_ and PNCs can also have different emission sources, particularly in urban environments^48^. Furthermore, the negative relationship indicates that at high particulate mass concentration, driven by large particles, the number concentration of small particles diminish due to coagulation and condensation (sink) processes.

Meteorological features are found in the middle range of the SHAP order. BLH appears as the most influential of the three, followed by temperature and precipitation. BLH had an inverse relationship with the model output, as the SHAP values were positive at low values. Kesti *et al*. have shown that when the BLH is low, particles are confined to a shallow mixing volume near the surface, thus, contributing to increased concentrations^69^. Moreover, a shallow boundary layer limits vertical mixing, trapping pollutants near the surface, and, conversely when BLH is high, particles disperse throughout a larger air volume, effectively reducing the surface concentrations^43,70^. Precipitation shows a similar tendency, as it affects PNC through wet deposition processes. Below cloud-scavenging, where falling rain droplets collect particles and remove them from the atmosphere and in-cloud scavenging, where particles and precursors gases are incorporated into cloud droplets and removed during subsequent precipitation events^44,71^. It was also shown that particle removal through wet-deposition is less important for long-term average concentrations than the mixing effects of the boundary layer^71^, in line with our findings in this study.

The last of the meteorological features indicates a mix of influences towards the model output, as it contributes both negatively and positively across its range. Low temperatures can enhance PNC values, as they promote condensation of semi-volatile compounds, reducing their saturation vapor pressure, while at the same time their evaporation is reduced.^72^. Conversely, during periods with relatively high temperatures and solar radiation intensity, photochemical oxidation of volatile species into less volatile ones promotes new particle formation and PNCs^20^. The relationship between temperature and PNC is complex and is often intertwined with other meteorological parameters, such as BLH and precipitation. These complex interactions can lead to different PNC responses depending on the local environment and emission sources^73^, which is reflected in the analysis of the SHAP values.

The relatively low importance of the road network feature (SHAP values below 0.025) seems counter-intuitive given that traffic is a major source of particles, especially in urban environments. However, this has several reasons. First, the impact of road traffic is already captured by other features in the model, particularly NO_2_ concentrations and black carbon emissions, both commonly used as proxies for traffic-related sources^74,75^. Second, the static nature of this feature may not fully capture the dynamic nature of traffic emissions, which vary significantly with time^76^.

Lastly, the weaker influence of static features like the road network compared to dynamic variables supports recent findings that emphasise the importance of temporal variations in emission patterns over fixed geographical features^75^. The weak SHAP influence of road networks contrasts with established traffic-UFP correlations but aligns with limitations in our modelling framework. First, collinearity between road density and traffic-related pollutants (e.g., NO_2_, BC) likely obscures the unique contribution of road networks, as these covariates act as proxies for traffic sources. For example, LUR models often report masking effects when multiple traffic indicators are included, with NO_2_ and BC absorbing explanatory power that might otherwise be attributed to road features^77^. Second, static road data inadequately capture temporal traffic dynamics, such as rush-hour congestion or seasonal freight activity, which drive short-term UFP spikes. Studies highlight that static road metrics (e.g., annual road density) fail to reflect real-time traffic volume or fleet composition (e.g., diesel vs. electric vehicles), weakening observed correlations^78^. Additionally, low spatial variability in road density across regions (e.g., uniform distributions in suburban/rural grids) reduces discriminatory power, a common issue in LUR models relying on coarse road datasets^79^.

**Fig. 8:**
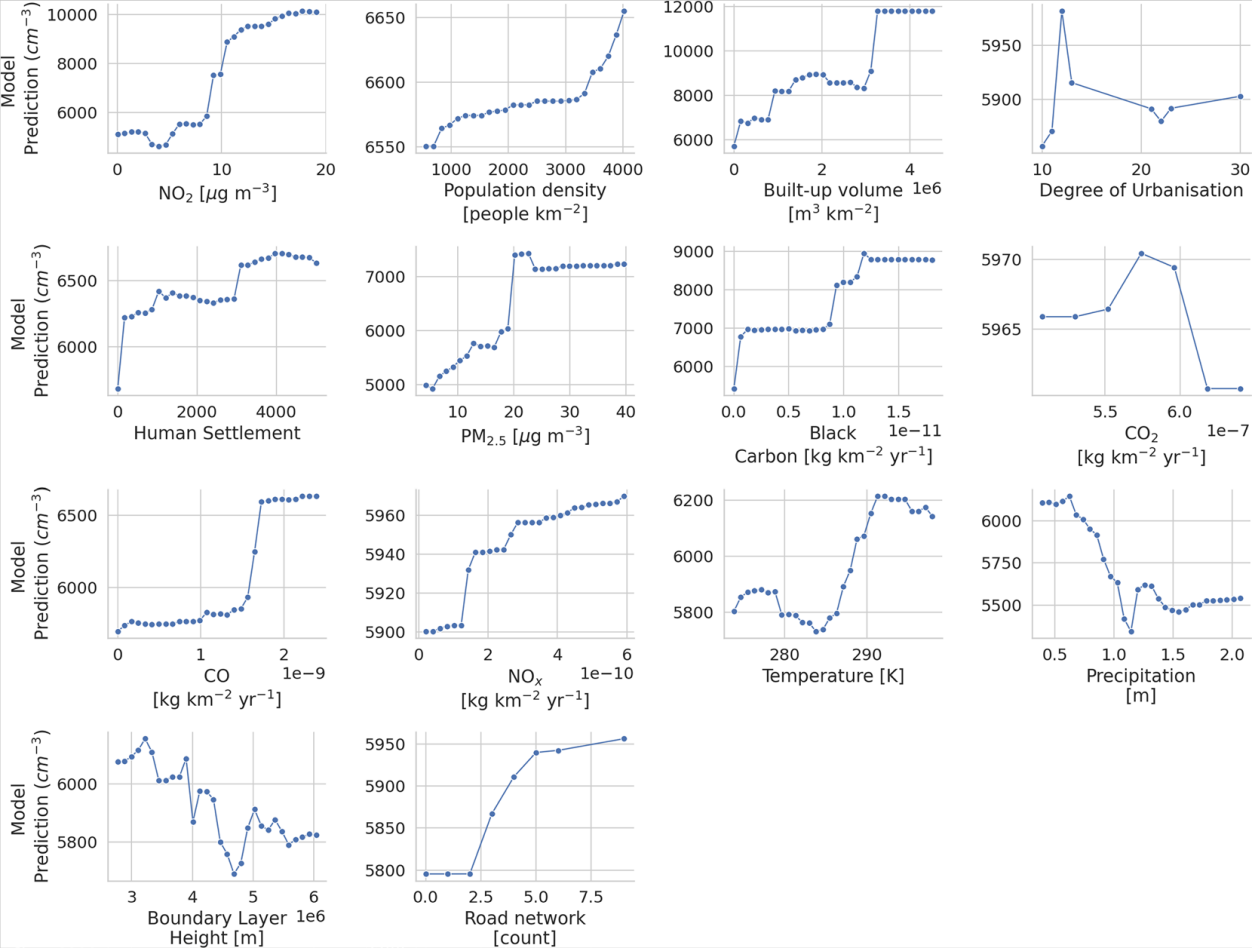
Partial dependence plots (PDPs) for the set of input variables used in the PNC prediction model. The PDPs show the marginal effect of each feature on predicted PNC while holding the rest of the features at their mean value.

To further investigate the marginal effect of each feature on model predictions and to complement the SHAP analysis, we provide partial dependence plots (PDPs) for all predictors (Fig. 8). PDPs illustrate the relationship between individual predictors and the predicted PNC, marginalizing over the distribution of other variables and thereby enabling a more direct interpretation of the model’s dependence structure^80^. The PDP for the road network variable supports its relatively weak effect on model output, consistent with the low SHAP values observed in the beeswarm plot. In contrast, features such as *NO*_2_, built-up volume and BC clearly exhibit stronger, monotonic, or non-linear influences on PNC predictions, in line with both domain knowledge and their high SHAP importances. This further supports our interpretation that multicollinearity, especially between road network, *NO*_2_, and BC (all proxies for traffic emissions), dilutes the apparent unique contribution of the road feature in the presence of more temporally-resolved variables.

### Sources of uncertainty

In this study we applied a novel data-driven methodology to predict PNC on a global scale at high spatial resolution. Our predictions are, however, subject to multiple sources of uncertainty that need to be carefully considered. These uncertainties can be broadly categorised into data-related, model-related, and prediction-uncertainties.

#### Data uncertainties

The primary source of data uncertainty includes the limited spatial coverage of ground station measurements, particularly in regions with significant pollution sources. The sparse distribution of PNC monitoring stations, especially in low- and middle-income countries with growing air pollution from industrial activity and urbanization, introduces sampling uncertainty. This limitation is particularly notable in regions like Africa, South America and parts of Asia, where data availability remains sparce despite their significant contribution to global emissions. Most of our training data originates in Europe and North America, with only a limited number of cities represented in Asia. Other regions, especially in the Southern Hemisphere remain under-represented in our training dataset.

While we do not expect this to be a major limitation for urban locations—since the PNC and UFP data and features included in our analysis span a wide range of environmental conditions, including diverse climates, emission densities, land use types, and population densities (as quantified in the partial dependence plots (Fig. 8), where the x-axis represents the central 95% interval of each variable’s observed range), it will nevertheless be important to further test the model’s performance as new measurement data become available in currently underrepresented regions. Additionally, even though spatial cross-validation partially addresses the issue of spatial bias, it cannot fully mitigate the risk of extrapolation errors in regions with sparse or absent ground measurements. The predominance of European and North American data in our training set means that model predictions for under-represented regions should be interpreted with caution, as the model may not fully reflect the local emission profiles or environmental conditions unique to such areas. This limitation highlights the urgency for expanded monitoring networks, targeted data collection in under-sampled regions and open-access sharing of such data to enhance model generalizability and reduce uncertainty in global exposure assessments^81^.

#### Model-related uncertainties

The XGBoost model provides reliable predictions through its ensemble structure and regularization mechanisms. Its demonstrated robustness in cross-validation tests—particularly in data-scarce regions—reflects an efficient balance between model complexity and generalizability. Spatial leave-one-out cross-validation (LOOCV) results show the framework adapts to environmental heterogeneity by widening prediction intervals in regions with limited training data, a critical feature for global-scale applications^82^. XGBoost’s performance on modestly sized datasets aligns well with the available ground observations, as its gradient-boosted trees capture nonlinear relationships without overfitting to sparse or noisy measurements^83^.

While the spatial LOOCV R^2^ of 0.77 indicates a reduction in model performance when extrapolating to regions outside of the model’s training set, this level of accuracy is consistent with previously published exposure assessment models used in epidemiological research. For example, land-use regression (LUR) models applied in multi-city studies often report spatial LOOCV R^2^ values in the range of 0.5 to 0.8, yet have been successfully used to detect significant health impacts, including respiratory and cardiovascular outcomes, in both urban and rural settings^77,79^. Similarly, recent hybrid models for PM_2.5_ and NO_2_ have achieved comparable R^2^ values in data-scarce regions, supporting their use in global and regional health burden assessments^63,84^.

Nevertheless, the challenge of extrapolating to regions with limited or no ground-based monitoring remains a key limitation for all global-scale models. Approaches such as hybrid modelling^84^, which combine machine learning with process-based chemical transport models, and the use of satellite-derived proxies and low-cost sensor data^63^, can improve predictions in under-monitored areas. Our model’s integration within a conformal prediction framework further allows for the detection and flagging of regions with expanded coverage intervals, where the model underperforms and where exposure estimates should be interpreted with caution.

#### Prediction Uncertainties

Prediction uncertainties arise from both aleatory uncertainty (inherent variability in the system) and epistemic uncertainty (lack of knowledge). These uncertainties are most pronounced when predicting PNCs in regions with environmental conditions significantly different from the training data, i.e., when extrapolating to areas with limited ground measurements, and when dealing with temporal variations not well represented in the training dataset.

The conformal prediction framework we employed provides uncertainty quantification that accounts for these various causes, offering reliable coverage intervals without assuming a normal distribution of the data. The framework’s coverage ensures our uncertainty estimates remain valid even when the model accounts for new environmental conditions.

The varying performance across different population density classifications reflects how these uncertainties manifest differently in various environments. The model performance varies across different environments, as evidenced by the spatial LOOCV results (R^2^ = 0.77), indicating higher uncertainty in regions with limited training data compared to random cross-validation (R^2^ = 0.91). While urban areas show the lowest relative uncertainties, likely due to better representation in the training data, rural areas exhibit higher percentage errors, though this is less critical for exposure assessments given both the lower population density and PNC values in these regions.

Given these limitations, there is a clear need to expand the spatial and temporal coverage of PNC and UFP measurements across geographical regions and ensure their availability through open-access data repositories. This will enable the development of more comprehensive models and improve the reliability of predictions, particularly in currently under-monitored regions.

### User Guide

The dataset is provided in CF conventions compliant NetCDF format (.nc), a widely used, self-describing format for multidimensional datasets. Each NetCDF file contains annual mean PNC values, 95% coverage intervals, and approximated UFP values for 2010-2019, organized by latitude and longitude. Metadata describing variables, units, and conventions are included in the file and can be viewed with any of the recommended tools. Several free tools can be used for accessing NetCDF datasets, such as the *xarray* library in Python, the Panoply visualisation tool, QGIS, and the *ncdf4* and *raster* libraries in R.

## Data Availability

The global annual particle number concentration (PNC) and ultrafine particle (UFP) dataset generated in this study is available in open access from Zenodo at 10.5281/zenodo.14832351. The data are distributed in NetCDF format, with separate files for each year from 2010 to 2019. Each file contains gridded annual mean PNC values (PNC variable), estimated UFP values (UFP variable), and 95% coverage intervals (CI variable), indexed by latitude and longitude. The dataset is licensed under the Creative Commons Attribution 4.0 International License (CC BY 4.0).

## Code availability

The code used to produce the datasets presented in this study is freely and openly under an MIT license at https://github.com/pantelisgeor/Ultrafine-Particles and 10.5281/zenodo.14832351^61^.
